# Could Relatedness Help Explain Why Individuals Lead in Bottlenose Dolphin Groups?

**DOI:** 10.1371/journal.pone.0058162

**Published:** 2013-03-13

**Authors:** Jennifer S. Lewis, Douglas Wartzok, Michael Heithaus, Michael Krützen

**Affiliations:** 1 Florida International University, Department of Biological Sciences, Miami, Florida, United States of America; 2 Florida International University, Biscayne Bay Campus, Department of Biological Sciences, North Miami, Florida, United States of America; 3 Evolutionary Genetics Group, Anthropological Institute and Museum, University of Zurich, Zurich, Switzerland; Texas A&M University-Corpus Christi, United States of America

## Abstract

In many species, particular individuals consistently lead group travel. While benefits to followers often are relatively obvious, including access to resources, benefits to leaders are often less obvious. This is especially true for species that feed on patchy mobile resources where all group members may locate prey simultaneously and food intake likely decreases with increasing group size. Leaders in highly complex habitats, however, could provide access to foraging resources for less informed relatives, thereby gaining indirect benefits by helping kin. Recently, leadership has been documented in a population of bottlenose dolphins (*Tursiops truncatus*) where direct benefits to leaders appear unlikely. To test whether leaders could benefit indirectly we examined relatedness between leader-follower pairs and compared these levels to pairs who associated but did not have leader-follower relationship (neither ever led the other). We found the average relatedness value for leader-follower pairs was greater than expected based on chance. The same was not found when examining non leader-follower pairs. Additionally, relatedness for leader-follower pairs was positively correlated with association index values, but no correlation was found for this measure in non leader-follower pairs. Interestingly, haplotypes were not frequently shared between leader-follower pairs (25%). Together, these results suggest that bottlenose dolphin leaders have the opportunity to gain indirect benefits by leading relatives. These findings provide a potential mechanism for the maintenance of leadership in a highly dynamic fission-fusion population with few obvious direct benefits to leaders.

## Introduction

Group travel can be directed or suggested by a subset of individuals, generally referred to as leaders [1]–[5]. Followers in these groups can presumably benefit from the leader(s) locating otherwise unavailable resources or from being led to available ones more efficiently. When leaders lead from a vanguard position, leaders can gain direct benefits through “finders share” [6], [7] or by obtaining priority access to food finds if leaders are at the top of a dominance hierarchy [8]. However, when resources are mobile and patchy, it is just as likely that followers will locate resources at a time similar to the leader, and “finders share” or priority access benefits may be negligible. Under conditions where food is individually located (e.g. non schooling fish), cannot be subdivided or shared, and direct competition with other groups is unlikely, recruitment of others will not increase the foraging gains of leaders.

If finders share is negligible, immediate benefits (e.g. locating resources first) are likely not an impetus to leading. In fact, increased competition may be a cost of leading. For example, followers may reduce a leader’s intake when resources are located initially or through learning the location of resources that followers can exploit later. Potential cognitive load demands could also be a cost (leaders attention towards resource location reduces attention elsewhere [9]). For example, less time may be available to watch for predators (of concern particularly in small groups, where individual vigilance is still important). Given the lack of obvious immediate benefits and the certain costs to leading, indirect benefits created by helping kin locate resources may provide reason for individuals in fission-fusion groups to lead others.

Seemingly altruistic behaviors can occur when the provider gains a less obvious benefit (e.g. inclusive fitness [10], [11]). These acts may be subtle, such as lowered aggression [12], increased cooperation [13] or allowing for shared space use such as partial home range overlap [14]. This type of behavior can also appear more overt, where costs to the provider and benefits to the receiver may be more apparent. Examples include helping to care for young that are not direct progeny [15], [16] or forming alliances to aid with the procurement of resources [17]. In each of these cases, the providing group members are related to the recipient, and the providers may benefit indirectly if they improve the short and long term success of kin. Leaders in fission-fusion groups may lead relatives by deciding which groups to join and by choosing whether or not to participate in leadership behavior when they become a member of a group. If followers gain benefits from this interaction (e.g. if leaders have more knowledge about where to find areas with higher prey availability or how to efficiently and safely navigate to these areas), and leaders do not gain direct benefits in return, then the leader-follower relationship may also appear altruistic.

**Figure 1:**
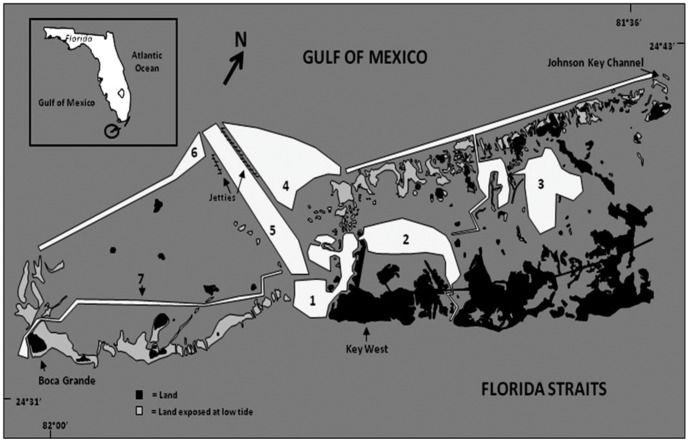
Lower Florida Keys research area. Numbered zones within the study area include all navigable waters.

To test if leaders in groups may lead relatives, we examined a population of bottlenose dolphins, *Tursiops truncatus*, in the Lower Florida Keys (LFK, Figure 1) where evidence for leadership had been documented [18]. We estimate that this population is small based on catalog size (current = 217) and the low frequency of new sightings (2% increase over past 111 group sightings). Bottlenose dolphins of the Lower Florida Keys exhibit highly dynamic fission-fusion grouping patterns with membership usually changing frequently (mean = 54±23 min SD between changes in group composition; unpublished data) and travel in small sized groups (mean = 4.4±3.3SD [19]). Teleosts are the most important prey of dolphins in the LFK study area. Fish are patchily distributed primarily due to high abundances associated with shallow (<3, water depth) seagrass beds that exhibit a patchy distribution [20]. Pinfish (*Lagodon rhomboides*) a solitary species, is the most abundant teleost. Other species such as lane snapper, (*Lutjanus synagris*) and grunts (*Haemulidae* sp.), which occur singly or in small groups, are also common (J. Lewis unpublished data). These species are all prey of LFK bottlenose dolphins [21] (Lewis personal observation). Because of the ephemeral nature of prey resources in the LFK (patchy distribution with no large schools encountered), bottlenose dolphins in the area do not socially forage (e.g., rounding up balls of schooling fish [22]), but instead, travel together and forage individually on single fish that are located, (e.g. mud plume feeding [23]). Leaders in these groups therefore do not benefit from location of prey sources first, or by help of other group members to secure prey. We have documented that consistent leaders (those that led significantly more times across groups sampled than was expected via chance, see further definition of a leader below) lead groups to areas with greater prey availability compared to individuals that were not consistent leaders, and that they do this following more direct routes than other individuals [18]. If leaders are leading relatives to these better foraging areas, then the potential for indirect benefits (by helping kin profit) is possible. To investigate this potential benefit of leading in fission-fusion groups, we examined relatedness within leader-follower pairs in the LFK dolphin population and compared to associates who did not have leader-follower relationships (non leader-follower pairs).

### Ethics Statement

For all animal sampling the following steps/protocols were taken to minimize disturbance. When biopsy sampling, to lower the impact per individual, we 1) sampled from the area just below the dorsal fin (avoiding the head and the belly), 2) we compared each individual targeted to a catalog of previously sampled individuals to ensure double sampling did not occur and 3) we used modified bolts which only penetrated the skin and blubber. Additionally, all equipment that contacted animals was sterilized prior to use to reduce the chance for infection. Dolphins sampled were monitored post biopsy for behavioral reaction and to ensure healing occurred.

Behavioral data used to determine leadership were collected from a boat which was maneuvered to minimize disturbance (e.g. slow speed, moving parallel to dolphins) and behavior sampling was abandoned if disturbance was noted (e.g. continued loud exhalations or tail slaps seemingly resulting from our presence).

Sample/data collection and sample handling were conducted under the required permits for each country where work occurred. Samples and data were collected in the United States under National Marine Fisheries Service Permit No. 779-1633, National Marine Fisheries Service LOC No. 572-1639 and Florida International University Institution Animal Care and Use Committee (IACUC) No. 07-003. Samples were exported to Zürich, Switzerland under United States Convention on the International Trade of Endangered Species permit No. 09US203311/9 and Swiss Convention on the International Trade of Endangered Species permit No. 3531/08.

## Materials and Methods

### Biopsy Collection

Skin samples were collected from 36 animals (across 30 different encounters) during 2008 in the Lower Florida Keys study area (Figure 1) using a recurve crossbow (Barnett Wildcat III) with modified bolt tips [24]. Tissue samples were stored in DMSO (20%) until analysis.

### Mitochondrial DNA Analysis

Total genomic DNA was isolated from skin samples using Gentra Puregene DNA Extraction Kit (Qiagen). Five hundred base pairs (bp) of the mitochondrial control region were amplified via Polymerase Chain Reaction (PCR) using primers dlp1.5 and dlp5 [25]. The reaction mixture (total reaction volume = 20 µl) included 0.6 µl of each primer, 0.4 µl dNTPs, 0.25 µl MgCl_2_, 2 µl buffer, 0.05 µl Taq polymerase (Sigma-Aldrich) and 1 µl of template DNA. The PCR profile began with denaturization at 94°C for 3 min, followed by 10 cycles of denaturization (30 sec), annealing (30 sec) and extension (1 min). Annealing temperature for these 10 “touch-down” cycles started at 63°C with a decrease of 1°C at each of the subsequent 10 cycles. This was followed by 21 additional cycles of 93°C for 30 sec, 52°C for 30 sec, and 72°C for 1 min with a final extension at 72°C for 1 min. Negative and positive controls were included in PCR runs and later used for validation of fragment amplification using 1.5% agarose gel electrophoresis. Polymerase chain reaction products were cleaned using GenElute PCR DNA Purification Kit (Sigma), and then both forward and reverse primers were run through a cycle sequencing reaction using a Cycle Sequencing Ready Reaction Kit (Applied Biosystems). Ethanol precipitation was used to purify the products from the cycle sequencing. Strands were sequenced using an ABI 3730 DNA Sequencer (Applied Biosystems). Sequencing Analysis 5.2 was used to edit the sequences manually which were then aligned using Lasergene SeqMan 7.0 (DNAStar). These sequences have been deposited in GenBank (Accession numbers:).

### Microsatellite Analysis

Each sample was genotyped at 26 loci (Table 1) using three Multiplex Polymerase Chain Reactions with labeled primers. The PCR’s were carried out for each multiplex using a reaction mixture of 1 µl of DNA template, 0.8 µl of Primer Mixture, 4.0 µl Master Mix (Qiagen) and ddH_2_O for a final reaction volume of 8.0 µl. The PCR thermal cycle for multiplexes one and two included initial denaturation at 90°C for 15 min, followed by 35 cycles of 95°C for 30 sec, 60°C for 90 sec, and 71°C for 45 sec. A final extension followed at 71°C for 2 min. Polymerase chain reaction products were sequenced on an ABI 3730 DNA Sequencer (Applied Biosystems). We determined allele size fragments using Gene Mapper 4.0.

**Table 1:** Primers used in three separate multiplexes for polymerase chain reactions.

multiplex 1			multiplex 2		multiplex 3		
Tur4_98^a^	Tur4_66^a^	Tur4_108^a^	D22^c^	D8^a^	Tur4_162^a^	MK9^b^	Tur4_153^a^
**Tur4_117** a	**Tur4_105** a		Tur4_138^a^	Tur4_141^a^	**Tur4_132** a	**MK5** b	**MK8** b
MK6^b^	**Tur4_128** a		**Tur4_91** a		**Tur4_80** a	**KWM12** d	MK3^b^
E12^a^	**Tur4_111** a		**Tur4_87** a		**Tur4_142** a	**EV37** e	

Primers in bold are those that provided useful results (i.e., successfully amplified, passed tests of Hardy Weinberg Equilibrium, linkage analysis and null alleles) and were used in relatedness analyses.

a [65],

b [66],

c [67],

d [68],

e [69].

Four loci (of the 26 original) were monomorphic for all individuals sampled so they were discarded from further analysis. Four others were also discarded after not meeting standards for frequency of null alleles (<0.05, Cervus 3.0). After sequential Bonferroni correction [26] the resulting 18 loci (shaded in Table 1) showed no deviation from Hardy Weinberg equilibrium and no linkage disequilibrium was observed (Genepop 4.0). We used these 18 loci to calculate pairwise relatedness coefficients (r_s_) [27] for all individuals sampled, excluding two that were progeny of known mothers that were also sampled. Progeny were excluded to avoid biasing our relatedness estimates. There were no identical genotypes used in this analysis. We conducted a rarefaction analysis using RE-RAT [28] to determine the minimum number of loci for accurate estimates of relatedness.

### Gender Determination

Gender was determined for all individuals sampled using a multiplex reaction [29]. We used a Polymerase Chain reaction (PCR) mixture of 1 µl of template, 0.3 µl of each primer (ZFX forward and reverse, and SRY forward and reverse), 0.2 µl dNTPs, 0.25 µl MgCl_2_, 2.0 µl buffer, 0.05 µl *Taq* polymerase, and 15.3 µl ddH_2_O for a final volume of 20 µl. Initial denaturization for 4 minutes at 94°C was followed by 34 cycles of 45 sec at 94°C, 45 seconds at 60°C and 60 seconds at 72°C and then final extension for 10 seconds at 72°C. We compared results to controls for a known male and female using gel electrophoresis (1.5% agarose).

### Relatedness between Leader-Follower Pairs

Previously, using vessel-based surveys supplemented with observations from a tethered airship, we determined that individual leaders could be identified in LFK dolphin groups by their presence in the front of a group both during surfacing events and subsurface travel [18]. Collecting data on individual positions was facilitated in our study area by small group sizes and a small population composed of individuals with easily distinguishable fin markings. Individuals in the front position were much more successful in initiating changes in the direction of group travel (i.e., change in heading ≥35°) than dolphins in other positions in the group [18]. Groups were defined as all individuals within approximately 100 m of one another that were likely interacting (i.e., traveling, foraging and socializing together). Between 2001 and 2007, leadership data were collected from 161 groups for ≥30 min [18] (mean = 87 min, SD = 50 min, range = 30–23 min [18]). These groups were stable in composition (i.e. no fission-fusion occurred during the leadership sampling period) and only a subset of individuals were in the lead position for ≥20% of the time each group was sampled, (46% of groups sampled had one leader, 44% had two leaders, 10% had three leaders, [18]). Because the majority of individuals (69%) within groups led <20% of the time, we considered group leaders as those who led ≥20% [18] for the following analyses. Followers were defined as group members who led <20% [18]. Groups tested for leadership ranged in size from 2 to 22 (mean = 5.1±3.3SD) when calves were excluded and 2 to 27 when calves were included (mean = 6.3±4.1SD) [18] (calves defined generally as ≤2/3 the size of the presumed mother and by presence near a specific adult female across multiple sightings). This group size is larger than the group size reported overall for our study area because it does not include sightings of lone individuals. Leadership in the LKF population is determined only during group travel (defined as continuous directional movement) between foraging bouts, and that this travel does not involve obvious social activity, such as herding females by males that has been observed in some populations of *Tursiops* sp. [30]. Herding activity where males separate females from other individuals and travel behind them has not been documented in the LFK bottlenose population.

Genetic data were available for 57 known leader-follower pairs. This data set of pairs included 11 different individuals as leaders and 20 as followers. Individual leaders were part of a mean of 5.18±SE 1.26 leader-follower pairs (range = 1–12 pairs). Individual followers were included in a mean of 2.85±SE 0.34 pairs (range = 1–6 pairs). All combinations were sampled only once (i.e., if pair A and B was noted with A as leader and B as follower, there were no pairings with B as a leader and A as a follower) (three of the possible pairs we had for analyses were discarded for this reason). Therefore the mean number of times an individual was listed as a leader or follower, also refers to the number of leaders or followers each individual paired with on average (i.e., followers were seen with on average 2.85 different leaders, range 1–6). Only adult pairs were used for this analysis (exact ages were not available). Adults and independent juveniles were defined as individuals who were sighted without constant association with a presumed mother during the time of data collection (i.e. both individuals were not in the same group for every sighting of those individuals). It is likely that most of the followers were mature adults and not juveniles when we sampled them (≥90%) based on documented motherhood for females and large body size of males. We determined an average r_s_ value for these 57 pairs. This value was compared to the distribution of average r_s_ values created by running 1000 random permutations of the entire data set which included leader-follower pairs and non leader-follower pairs (pairs that were seen together in groups who had leaders but the pair in question never had a leader-follower relationship). Many individuals in a group never exhibited a pair wise leader-follower association (i.e. both were followers). For each permutation, 57 r_s_ values were randomly selected from the population of all associating pairs of individuals (leader-follower pairs, and non-leader follower pairs), an average r_s_ value was calculated and this value was then placed within the distribution which eventually included 1000 averages. This allowed us to determine if the average for leader-follower pairs was greater than expected via chance. A second Monte Carlo randomization test was used to determine whether the number of leader-follower pairs that shared mitochondrial DNA haplotypes was larger than expected based on chance (using 1000 permutations of random selections of 57 pairs of individuals that associated). If leader-follower pairs were more closely related, mitochondrial DNA haplotype comparisons could provide information about how individuals might be able to recognize at least maternal relatives. Relatedness for all pairs per specific group was not available for our analyses (due to the difficult nature of field biopsy sampling). Instead pairs examined were those for which we had recorded a specific relationship (leader-follower or non leader-follower pairs) across leadership sampling.

### Leader-Follower Relatedness and Association Strength

Association Indices (AI) were calculated for the LFK population using the Half Weight Index in SOCPROG [31] with the sampling period of a day. Individuals were almost never sighted more than once during a day and if so only the first group sighting of an individual was used. Because of the small size of groups and distinctive markings of fins, we were able to identify all group members in 92% of groups. For the remaining 8% of groups, only one group member was not identified. As with previous studies of cetaceans [32]–[34], we only included individuals in the analysis if they had been sighted ≥5 occasions. Strong associations between Lower Florida Keys dolphins occurred over relatively long time periods within our study and do not appear to be driven by intense, but short term associations. As example, for the pairs of individuals who were sighted with one another >5 occasions during the study period (2001–2007), 85% had an average number of days between pair re-sights of <20. These sightings ranged across a period of on average 141 survey days (SE = 4.1, range = 17–199) (from time first noted as a pair to the last time noted as a pair). To determine if the resulting association matrix was different from random, we calculated the coefficient of variation (CV) for the observed matrix, and compared this value to the CV’s generated from 20,000 random permutations of the data (shifting groups within samples) [31], [32], [35] again using SOCPROG. The observed matrix was considered non-random if >95% of the permuted matrices had CV values less than the CV from the observed matrix. Further testing for non-random associations (as suggested [36]) included examining the correlation value between the observed and randomly generated AI’s, and also testing if the value for S^2^×H was >5, where S is social differentiation and H is the average number of associations per individual [36]. For all leader-follower pairs, we tested the correlation between r_s_ values and AI’s using a Spearman Rank Test. The same test was used to examine non leader-follower pairs. To examine whether trends were influenced by maternal relatives associating, we used a logistic regression to ask whether r_s_ value or AI predicted sharing of haplotypes.

## Results

**Figure 2:**
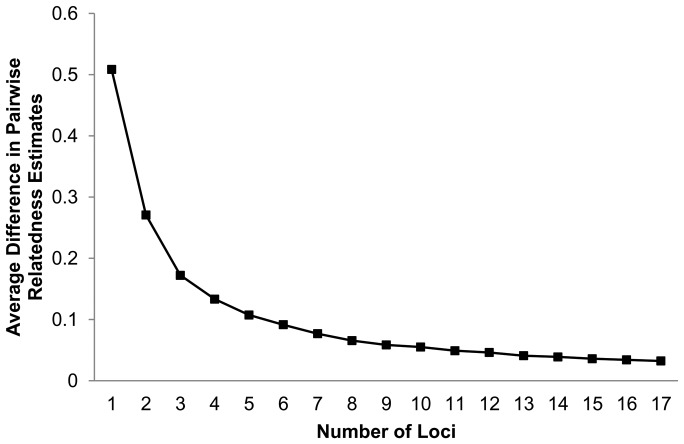
Differences in pairwise relatedness. Average differences in pairwise relatedness estimates [26] across 18 loci generated from rarefaction analysis using RE-RAT [27].

Leader-follower pairs had an average r_s_ value greater than expected based on chance (*P* = 0.009), while non leader-follower pairs did not (*P* = 0.96) (leader-follower pairs: mean r_s_ value = 0.09, *SE* = 0.02, non leader-follower pairs: mean r_s_ value = −0.03, *SE* = 0.02, mean r_s_ value of the random distribution = 0.016). We note that in tests of non leader-follower pairs, 59% of these pairs (48 of 81) included one individual who had been a leader in one of the 57 leader-follower pairs (10 of the 11 leaders pairing with between 2 and 9 other non-leader-follower associates). Non leader-follower pairs which contained a member who had led in one of the 57 leader-follower pairs, also did not have greater relatedness values than expected based on chance (*P* = 0.84) similar to our finding for all non leader-follower pairs (pairs including individuals who had led others and pairs without individuals who had led others). The rarefaction analysis revealed that there was a ≤0.04 average pairwise difference in relatedness values calculated using 13 loci, and ≤0.03 when using 16 loci (Figure 2), indicating that use of 18 loci provided accurate relatedness estimates.

Leaders, defined as those leading for ≥20% of the time in a particular group, included males (*n* = 6) and females (*n* = 5). Males led both males (n = 11) and females (n = 7). Females also led both genders (n = 21 males, 18 females). Seven mitochondrial DNA haplotypes were found among the sampled individuals. Only fourteen of the 57 leader-follower pairs (25%) shared haplotypes. The number of haplotypes shared between pairs of leaders and followers was not significantly more than expected based on chance (mean = 0.25, *SE* = 0.06, *P* = 0.34).

**Figure 3:**
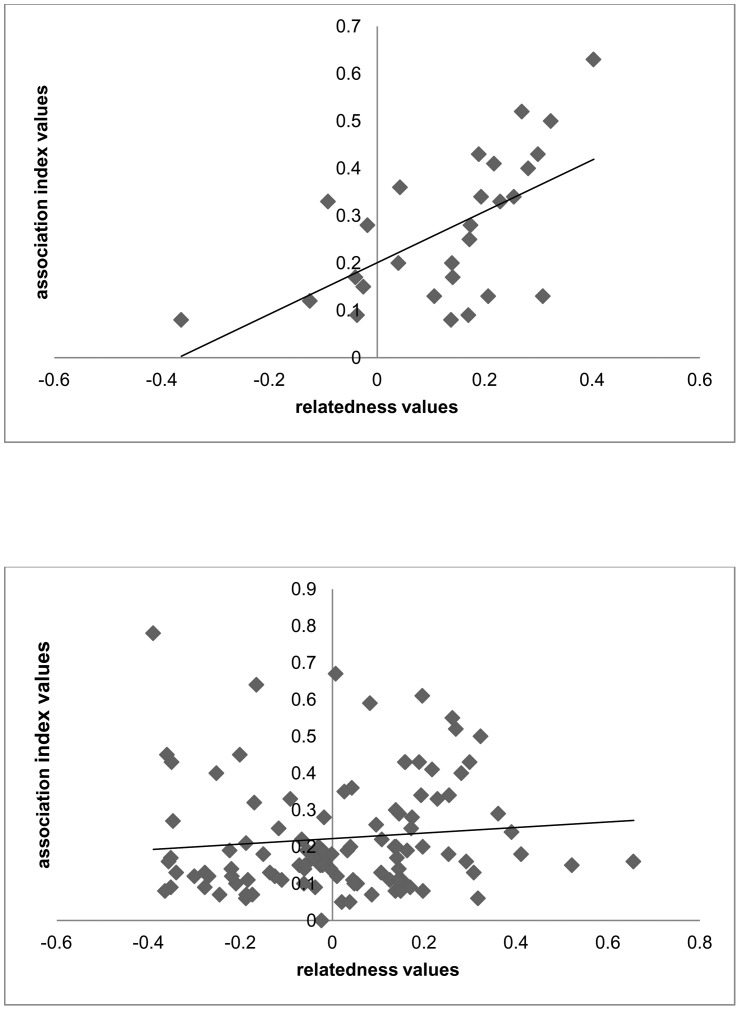
Association Index and relatedness values. Association Index values (Half-Weight Index [26]) plotted against relatedness values for (a) leader-follower dolphin pairs and (b) non leader-follower pairs in the Lower Florida Keys (leader-follower: *n* = 57, *r_s_ = *0.55, *P*<0.0001, non leader-follower: *n* = 81, *r_s_ = *0.07, *P = *0.51).

Our observed AI CV value, (*CV* = 0.77, *SD* = 0.16) was greater than expected via chance (*P*<0.01) (random generated *CV* = 0.75, *SD* = 0.01), indicating that associations in this population are not random. Additional evidence for non-random associations included an S^2^×H value >5 (1.06^2^×50.1 = 55.2) and *r* = 0.48. Association Index values were positively correlated with r_s_ values for leader-follower pairs (*r^2^* = 0.55, *P*<0.0001) (Figure 3a) but no relationship between AI and r_s_ value was found for non leader-follower pairs (*r^2^* = 0.07, *P* = 0.51) (Figure 3b). In other words, there was a tendency for leader-follower pairs who associated more frequently to have higher relatedness values while the degree of relatedness between individuals that did not lead or follow one another did not vary with the rate of association (i.e. high levels of association for non leader-follower pairs did not correlate with higher relatedness values). No relationship was found when using only the 48 pairs of associates where the pair had never had a leader-follower relationship but one pair member had been a leader of others (*r^2^* = 0.23, *P* = 0.08). Neither r_s_ value (*df* = 1, *X^2^* = 1.66, *P* = 0.19) nor AI (*df = *1, *X^2^* = 0.49, *P* = 0.82) predicted sharing of haplotypes between leader-follower pairs, indicating correlations found were not due to high levels of association between maternal relatives.

## Discussion

Our findings provide support for the hypothesis that leader-follower relationships are based on kinship. We found that leader-follower pairs were more closely related to one another than expected based on chance, and that association frequency for these pairs was correlated with relatedness. This differed from non leader-follower pairs whose associations were not based on kinship. Additionally, while some leader-follower pairs were maternal relatives, this was not the case for all leader-follower pairings.

Finding that leader-follower pairs were more closely related to one another than expected by chance was somewhat surprising because bottlenose dolphins exist within a highly dynamic fission-fusion system and have no obvious mechanism for determining paternal relatives (mating is promiscuous [37]). Still, strong associations for male alliance pairs have been linked to relatedness in the bottlenose dolphin [17], but this result is not universal [38]. Our results are particularly interesting because the lack of shared haplotypes found for LFK leader-follower pairs indicated that the leader-follower groupings in the LFK are not merely the result of always following maternal relatives. Close associations based on maternal relatedness have been found in other odontocetes, such as killer whales [39] and pilot whales [40]. However, these associations occur over much longer periods (years) than those among individual dolphins in the LFK (hours or days) where far more fission-fusion activity occurs. The low frequency of maternal associations found in our sample of leader-follower pairs suggests that individuals are associating also with paternal relatives. Examples do exist where individuals seem to have information about paternal relatives in other mammals. Avoidance of paternal kin for reproduction has been cited in other group forming species where kin and non-kin come into contact (e.g. yellow baboons, *Papio cynocephalus* [41]; mandrills, *Mandrillus sphinx* [42]; African elephants, *Loxodonta* sp. [43] and white-faced capuchins, *Cebus capucinus* [44]). Phenotypic matching through olfaction has been suggested as a method for paternal kin recognition in both terrestrial (e.g. African elephants [45]) and aquatic species (e.g. bluegill sunfish: *Lepomis macrochirus* [46]; three-spined sticklebacks: *Gasterosteus aculeatus* [47]), even when individuals were not raised together (e.g. golden hamster: *Mesocricetus auratus,* [48]). However, dolphins likely have little sense of smell due to reduction of olfactory nerves [49]. Phenotypic matching using vocal cues is possible for determining maternal kin in bottlenose dolphin groups. Kin recognition between mothers and calves using signature whistles has been demonstrated for bottlenose dolphins in Sarasota Bay, Florida, (i.e. mothers and offspring recognize one another [50]). This recognition can occur because of the time spent in close association during the first few years of the calf’s life (usually ≥2 years [37]). Recognition of the whistle characteristics between associates outside of the mother/calf has also been demonstrated in *Tursiops* sp. (e.g. whistle convergence between alliance males [51], [52], and patterning whistles after those of other community members [53]). These studies indicate that bottlenose dolphins have the capacity to recognize vocalizations (signature whistles) of individuals that they repeatedly come into contact with. The question is whether there is any mechanism that would allow individuals to use this information to recognize paternal kin.

Behavioral mechanisms that could be used for kin recognition include use of rules based on information gained through association rates [41, 54 and 55]. Patterns of association when linked with levels of relatedness may provide kinship cues (e.g. African elephants [43]). Frequency of associations between LFK bottlenose dolphins (even though under flux) may provide a rule of thumb mechanism to allow avoidance or association with relatives, at least for maternal siblings, because calves that are half siblings or cousins are likely to interact during the time of dependency (which can last up to four years and sometimes beyond [37]). It is possible that high rates of association with adult males may provide something similar for paternal relatives. For example, in some species (including bottlenose dolphins [37]) individuals may group according to reproductive state (e.g. mothers with calves [56]). If a subset of males have reproductive monopolization for >1 year over a large number of mating attempts, the chances of fatherhood being shared between calves of the same age group that are raised within the same group (or associating with the same mother/calf pairs frequently), will be more likely [55], [57]. This may be possible if wild bottlenose males exhibit some form of dominance hierarchies, as has been documented in captive groups [58]. If reproductive association occurs, and males continue to associate with the same females frequently after the calves are born, a rule of thumb for association could be developed as a method for a response that correlates with relatedness [41], [55] (i.e., higher associations = greater probability of maternal and paternal relatedness). In the LFK population, groups often include adult males and mother-calf pairs, and the frequency of associations with the same males is high. For example, four adult males were sighted with female 002 during 29–36% (depending on the particular male) of the 76 sightings for this female who had a calf ≤3 years of age at each sighting. This type of grouping, even though dynamic, may provide enough information to allow a rule-based system to use for behavioral avoidance or association with kin. Some evidence exists for frequent associations with biparental relatives in bottlenose dolphins [59], but no studies to date have specifically tested grouping associations and paternal relatedness. Further genetic study will be necessary to determine whether a small number of males do monopolize reproduction in the LFK population.

Other factors that could play a role in leader-follower associations in animal groups include age, size, reproductive state and gender. Social groupings of mammals can form according to these characteristics, (e.g. tendency for females to group and males to associate with others less often in sperm whales: [60], and African elephants: [61]). However, these characteristics do not appear to play a central role in determining leader-follower relationships in LFK bottlenose dolphins. Indeed adults and calves are present in most LFK groups (78% of groups with >1 individual, n total = 261 groups), as are both genders (87%, n = 129 of 149 groups with known genders), and females of varying reproductive states (i.e. with calves and without calves) (84% of groups with known females, n = 73 of 87 groups) (Lewis, unpublished data). In addition, leaders were of both genders, and both male and females leaders were followed by males and females. Although subtle differences in body size and age within adult-sized animals could not be determined, it is unlikely that these factors contributed to association patterns that we observed between leaders and followers. In other populations reproductive behavior may result in individuals controlling group movement from positions other than vanguard. For example, adult male dolphins in Shark Bay, Australia adopt positions behind receptive females with which they are consorting to minimize the chances for the female to escape [30]. Such herding behavior has not been observed in the LFK. Additional factors that could also be considered include population genetic structure, and dispersal. Unfortunately, we do not currently have data that would allow us to address the relative importance of these factors.

While no trends were apparent regarding the gender of leader-follower pairs, overall, females did have more followers than males. Although further work is needed to explore sex differences in the number of followers, previous studies in this population found that all individuals that were consistent leaders were female [18]. These results do indicate that while males and females lead, females may have greater influence within this population of bottlenose dolphins.

While the dynamics of forming leader-follower associations is still uncertain, it appears that for LFK bottlenose dolphins there is a tendency for leader-follower pairs to be more closely related, while no such relationship is found for non leader-follower pairs. In leader-follower pairs, r values increased with increasing AI values. This was not found for non leader-follower associates, including non leader-follower associates where one member of the pair had led others before. These findings indicate that not all types of associations are based on kinship in this population, but leader-follower associations can be. We note that due to the fission-fusion nature of grouping in bottlenose dolphins, there may be pairs of individuals in each group that vary in relatedness value from other pairs (from low to high). But for leader-follower pairs that associate more frequently, relatedness values are usually larger. Preferential associations with close relatives could occur because potential benefits could result from this association. LFK dolphin leaders may benefit by leading closer relatives to profitable resources (i.e., habitat with greater prey availability [20]) where these followers may have increased chances of locating food. Knowledge possessed by leaders may also provide greater safety to followers (e.g. avoidance of areas where stranding could occur, or where predation threat is larger). There are examples of animals providing guidance to resource use. In a *Tursiops aduncus* population in New Zealand, individual dolphins provide cues to stop and start feeding in response to potential knowledge of area depletion [62]. It has also been suggested that leadership by matriarchs in African elephant groups could provide aid towards resource location learned many years prior [63]. Because bottlenose dolphins are a fission-fusion type species, they have the ability to choose associates. Individuals can choose to be solitary, follow (by allowing others to control movement choice and falling behind), or to lead others (by making movement decisions when other individuals are willing to follow). Making these decisions more often in the presence of specific individuals (closer relatives) indicates that benefits to these leaders should result from these interactions.

Whether benefits to leaders gained by helping relatives are necessary for the evolution and maintenance of leadership within a highly dynamic fission-fusion population remains to be tested. It may be that conditions required for this behavior are relatively rare. While leadership when traveling has been suggested anecdotally for some other fission-fusion species (e.g. African elephant [63], spider monkey; *Ateles* sp. [64]) outside of the LFK dolphins, quantitative data to demonstrate consistent leadership when traveling in highly dynamic fission-fusion populations is scarce. In the case of the LFK dolphins, while indirect benefits of helping relatives may be important to leaders, environmental conditions of the area may play a larger role in shaping leadership behavior patterns for these animals. The LFK habitat is heterogeneous (i.e., multiple basins divided by impassible shallows and mangrove islands with limited entry and exit points) and requires knowledge for efficient and safe exploitation of resources [18]. If leaders have greater habitat knowledge, this may provide an important benefit to followers.

Our results provide insights into the development of leader-follower behavior when traveling in a highly dynamic fission-fusion species. Where resources are mobile and patchy, vanguard positional leadership should not result in increased access to prey. However, if specific individuals have greater habitat knowledge gained through experience, they can provide a source of information to those that follow them. Providing this resource could benefit relatives directly and therefore leaders indirectly.

To determine the relative importance of environment, kinship and social constraints in driving the occurrence and prevalence of leadership, comparisons with other populations of bottlenose dolphins under various environmental conditions (heterogeneous vs. homogenous) and of other taxa with various levels of group stability will be necessary. Additionally, documentation of whether benefits (e.g. increased foraging opportunities or avoidance of areas that pose a threat) are gained from following specific individuals will be important. Understanding the conditions where leadership may develop (particularly within a fission-fusion social system) will provide further insight into the benefits of group formation and the importance of specific individuals in group success.
